# Examining techniques for measuring the effects of nutrients on mental performance and mood state

**DOI:** 10.1007/s00394-015-1143-3

**Published:** 2016-01-07

**Authors:** Mark Hamer, Louise Dye, E. Siobhan Mitchell, Sophie Layé, Caroline Saunders, Neil Boyle, Jeroen Schuermans, John Sijben

**Affiliations:** 1 Department Epidemiology and Public Health, Psychobiology Group, University College London, 1 - 19 Torrington Place, London, WC1E 7HB UK; 2 Human Appetite Research Unit, Institute of Psychological Sciences, University of Leeds, Leeds, UK; 3 Nestlé Institute of Health Sciences, EPFL Campus, Lausanne, Switzerland; 4 Nutrition et Neurobiologie Intégrée, UMR 1286, INRA, 33000 Bordeaux, France; 5 Nutrition et Neurobiologie Intégrée, UMR 1286, Université Bordeaux, 33000 Bordeaux, France; 6 PepsiCo, 450 South Oak Way, Green Park, Reading, RG2 6UW UK; 7 Lucozade Ribena Suntory Ltd, 2 Longwalk Road, Stockley Park, Uxbridge, UB11 1BA UK; 8 ILSI Europe, Brussels, Belgium, International Life Sciences Institute, Europe, 83 Avenue E. Mounier, B6, Brussels, BE 1200 USA; 9 Nutricia Research, Nutricia Advanced Medical Nutrition, Utrecht, The Netherlands

**Keywords:** Mood, Cognition, Objective, Subjective, Food, Mental health, Affective assessment

## Abstract

**Purpose:**

Intake of specific nutrients has been linked to mental states and various indices of cognitive performance although the effects are often subtle and difficult to interpret. Measurement of so-called objective variables (e.g. reaction times) is often considered to be the gold standard for assessing outcomes in this field of research. It can, however, be argued that data on subjective experience (e.g. mood) are also important and may enrich existing objective data. The aim of this review is to evaluate methods for measuring mental performance and mood, considering the definition of subjective mood and the validity of measures of subjective experience.

**Methods:**

A multi-stakeholder expert group was invited by ILSI Europe to come to a consensus around the utility of objective and subjective measurement in this field, which forms the basis of the paper. Therefore, the present review reflects a succinct overview of the science but is not intended to be a systematic review.

**Results:**

The proposed approach extends the traditional methodology using standard ‘objective’ measurements to also include the consumers’ subjective experiences in relation to food. Specific recommendations include 1) using contemporary methods to capture transient mood states; 2) using sufficiently sensitive measures to capture effects of nutritional intervention; 3) considering the possibility that subjective and objective responses will occur over different time frames; and 4) recognition of the importance of expectancy and placebo effects for subjective measures.

**Conclusions:**

The consensus reached was that the most informative approach should involve collection and consideration of both objective and subjective data.

## Introduction

Nutrition plays an important role in brain function throughout the entire lifespan. Appropriate nutrient intake allows normal brain growth and neurodevelopment in early life and reduces the risk of cognitive decline and Alzheimer’s disease in older age [1, 2]. Our knowledge about nutrition has greatly evolved over the last 50 years, and in developed countries, food is no longer seen uniquely as a survival necessity and a primary need, but is associated with promotion of mental fitness [3–5]. The effects of nutrients on cognitive performance—mental processes (e.g. attention, memory, reasoning, problem solving, decision-making) related to the processing of information and application of knowledge—have been widely examined usually by employing objective outcomes measures such as reaction time and accuracy. The effects of nutrition on subjective measures of psychological functions, such as self-reported mood, have received relatively less attention. The aim of this review is to provide a comprehensive overview of the theoretical frameworks that link subjective experience of mood to cognition and, from this understanding, identify the most promising methods to measure the individual experience of mood and its relationship to cognitive function. This will be illustrated with examples from studies that have examined the effects of nutritional interventions on cognitive performance and mood. However, the effects of nutritional manipulations are often subtle and inconsistently reported (e.g. Halyburton et al. [6]; Hellhammer et al. [7]; Rogers et al. [8]). Therefore, this review will also draw upon examples from the stress, cognition and mood literature, since stress induction [e.g. the use of sensory overload (excessive noise) or performing a task under social evaluation to provoke a psychophysiological stress response] is both robust and reliable in the provocation of mood change and moderation of cognitive function [9, 10]. Whilst stress response can be termed as any alteration to homoeostasis in an organism, for the purposes of this review, stress is defined as a mismatch between the demands of a task/context/stimuli and perceived ability to cope. If a context/stimuli is perceived as stressful, this will provoke a subjective response (e.g. negative affect/mood) and a physiological response (e.g. the release of the hormone cortisol to orchestrate the neuroendocrine response to stress). The main topics that will be addressed are: (1) how to best measure mental performance and mood, i.e. which measures are most reliable and validated under which conditions, (2) how placebo effects may affect subjective state and response to nutrient intervention, (3) the relationship between objective and subjective measures, (4) the ability to detect meaningful effects and elucidate their mechanism of action and (5) how nutrient effects on subjective experience, measured by standardised questionnaires, translate into everyday life and the clinical relevance of these. The proposed approach extends the traditional empirical approach of using solely standard “objective” measurements to also consider subjective state in a more integrated manner using advances in technology and appropriate analytical strategies. Such data on subjective experience may enrich our understanding and interpretation of objective measures and as such these recommendations are expected to help guide researchers from academia and industry when studying the effects that foods or specific product consumption can have on mood and mental performance.

## Definition and measurement of subjective mood state

In behavioural sciences, measures of motor and cognitive behaviour (reaction time, number of words recalled) and physiological variables (salivary cortisol concentration, blood pressure, heart rate variability) are considered to be objective measures, in contrast to self-rating/self-reported measures of cognitive performance (e.g. participants’ self-rated perception of how accurately or quickly a task was performed) and mood, which are often referred to as subjective measures of an individual’s subjective experience. Mood can be described as a pervasive and predominant affective state and is commonly conceived to vary along orthogonal and bipolar dimensions of valence (positive vs negative) and arousal/activation (e.g. Russell [11]; Watson and Tellegen [12]). Mood can also be temporally separated into protracted (e.g. depressed mood) and transient, fluctuating affective states (e.g. a momentary state of increased vigour). As mood is inherently phenomenological, it is perceived as an inconsistent measure of the brain’s output, despite demonstrations that mood can be consistently measured. For example, specific drugs (e.g. Brown and Chandler [13]; Mula and Sander [14]) and stimuli (e.g. stress [15]) have been shown to have very consistent effects on mood, as measured by ratings on scales and questionnaires. In addition to well-being questionnaires, which often capture protracted, generalised affective mood states, there are several validated tools available to assess transient mood states which have been able to capture changes in mood as a result of a number of different nutritional interventions [16, 17]. For example, relative to placebo, a 30-day high-flavanol chocolate drink improved self-rated calmness and contentedness, measured by Bond–Lader mood scales [16] and the Profile of Mood States (POMS) captured changes in tension–anxiety scores following intervention with L-theanine under conditions of induced stress [17]. Table 1 provides examples of questionnaire/scale measures of subjective mood state and well-being and their potential suitability for nutritional interventions.

**Table 1:** Examples of measures of subjective mood state and well-being

Measure	Scales	Strengths and weaknesses	Reliability (Cronbach’s alpha [*rα*])
Bond–Lader visual analogue scale (BL-VAS) [69]	Subscales for calmness (encompassing individual questions on the extent of relaxation or calmness), contentedness (encompassing the emotions contented, tranquil, happy, amicable and gregarious) and alertness (encompassing alert, strong, well-coordinated, energetic, quick-witted, attentive, proficient, interested and clear-headed)	Using specifically selected subscales of common mood states, such as calmness and contentedness, it fails to capture a wide spectrum of other emotions, such as anger, or mind states that might not be viewed as strictly emotion, such as confusion or guilt	All scales: *rα* = .77–.93 [70]
The Positive and Negative Affect Schedule (PANAS) [71]	Ten positive affects (interested, excited, strong, enthusiastic, proud, alert, inspired, determined, attentive, and active) and ten negative affects (distressed, upset, guilty, scared, hostile, irritable, ashamed, nervous, jittery, and afraid)	When measuring moods after food consumption or relating to nutrition, such diverse emotions as hostility or pride may not be useful. Simply asking such questions can induce differential emotions	All scales: *rα* = .85–.90 [71]
The Profile of Mood States (POMS) [72]	A measure of six dimensions of transient and distinct mood states. The subscales are: tension/anxiety, vigour/activity, depression/dejection, fatigue/inertia, confusion/bewilderment, and anger/hostility. A total negative mood score can also be computed by adding up the five negative mood subscales and subtracting the positive scale vigour/activity	Widely used in cross-sectional studies and found to discriminate between different habitual diets; used in acute and chronic studies of intake of specific nutrients, vitamins, minerals and herbal supplements	Subscales: *rα* = .63–.92; Total score: *rα* = .75–.92 [73, 74]
World Health Organization Quality of Life Scale [75]	Comprises 26 items, which measure the following broad domains: physical health, psychological health, social relationships, and environment	Unlikely to pick up acute effects of food, but might reflect influence of habitual dietary patterns	Physical domain: *rα* = .82; psychological domain: *rα* = .81; social domain: *rα* = .68; environmental domain: *rα* = .80 [76]
The Satisfaction with Life Scale (SWLS) [77]	Comprises five items on satisfaction with life	Retrospective life experiences, may not be directly related to effects of diet	All scales: *rα* = .61–.81 [77]
Subjective Happiness Scale (SHS) [78]	Four-item scale of global subjective happiness. Two items ask respondents to characterise themselves using both absolute ratings and ratings relative to peers, whereas the other two items offer brief descriptions of happy and unhappy individuals and ask respondents the extent to which each characterisation describes them	Measures only one dimension of mood, namely positive feelings	All scales: *rα* = .74–.94 [78]
General Health Questionnaire (GHQ) [79]	Four subscales consisting of somatic symptoms, anxiety/insomnia, social dysfunction, and depression. (Multiple versions differing in number of items: GHQ-60, 30, 20, 28. 12)	Validated as a tool to assess mental health in large population samples; symptoms may be enduring and less likely to vary in relation to transient nutritional induced changes in subjective state	All versions: *rα* ≥ .80 (e.g. Dale et al. [81], Carvalho et al. [80], Montazer et al. [82])

Moods can be linked to specific events, such as those caused by artificial mood inductions, but more commonly they occur somewhat independently of second-by-second, or minute-by-minute circumstances. Ecological momentary assessment (EMA) or experience sampling methods (ESM) are alternative mood measurement tools which may more realistically capture sequential, transient states by requiring brief inputs of mood state during daily activities. EMA data can be captured, for example, via mobile devices such as smart phones, where participants are periodically asked to fill in short questionnaires. Psychological research has established that recollected effect may diverge from actual experience because it is influenced by errors in recollection, recall biases, focusing illusions and salient memory heuristics [18]. This “memory–experience gap” between life as it is remembered and life as it is experienced may be important to the processes through which the past impacts on future behaviour. Moreover, effects resulting from interventions with nutrients may not be noticeable after acute administrations commonly applied in laboratory settings, but may give rise to improved well-being over time, especially if the intervention has other benefits such as cardiovascular health. One recent study, which endeavoured to measure daily fluctuations in mood via cell phone questionnaires, reported an effect of a multivitamin supplement on alertness after 4-week supplementation [19]. Others have validated cardiovascular biomarkers against the mood reports acquired during daily life. For instance, Steptoe and colleagues found that systolic blood pressure correlated with positive effect in men during work hours [20].

## Validity of measures of subjective states

There are numerous types of validity, but in this context, the most important is construct validity—are we actually measuring what we think we are measuring? Evaluation of construct validity involves demonstrating that the measure correlates with variables that are known to be related to the construct (purportedly measured by the instrument being evaluated or for which there are theoretical grounds for expecting it to be related). Thus, identifying the “gold standard” against which to validate is crucial. Employing the stress induction model to illustrate this point, the seminal work of Seyle [21] explained that many stressors placed upon us have neutral effects until, by our own thinking, we appraise the experience as subjectively negative or positive. Thus, when measuring stress in individuals, it is important to acknowledge that a self-report of stress will need to be evaluated against the baseline of an individual (i.e. personality, past experience, as well as physiological state). This explains why different people may have profoundly different reactions to the same basic source of stress. In other words, it is one’s own perception that is crucial. However, both physiological and subjective mood responses to stress are relatively stable individual traits and can thus be accurately and consistently measured after exposure to stressful stimuli. For example, good correlations between subjective and physiological stress increases are shown in participants after the Trier Social Stress Test (TSST) [15]. Yet measurement of stress elicited by the TSST can be modulated by genetics, lifestyle, etc., and thus, validity of any subjective measurement will need to be evaluated in the context of these types of variables.

Contextual information is often missed when comparing measures; thus, validation of self-report with performance or physiological data must take into account large individual differences and also must control for as many external variables as possible. For example, measuring physical activity with objective (accelerometry) compared with self-report measures, not surprisingly produces very poor correlations [22] and is interpreted as reflecting recall bias inherent in self-report measures. However, an alternative explanation is that the two methods measure different things. Self-reports take into account the specific context, whereas accelerometry is a raw measure of movement. In summary, validity of subjective measures can be obtained via consistency of results after highly controlled stimuli such as the TSST and to some extent with physiological measures, when carefully controlled for “individual” factors.

## Detection and interpretation of meaningful effects

A common problem in this field is how to interpret null effects. Detecting no effect on a measure of subjective state following a nutritional intervention could mean that the intervention really does not influence the mood parameter measured. Alternatively, it could reflect a lack of sensitivity of that measure to small or subtle changes in state or that the intervention prevented a degradation that would otherwise have been observed. An effect may also fail to be detected because of inadequate power which is also related to the magnitude of the experimental effect, or individual differences in the use of a scale which increase variability and decrease sensitivity of the measure [5]. Large inter- and intra-subject variability in subjective measures such as mood mean that larger sample sizes to detect the effects of a nutrient against this relatively noisy background may be required. Null or inconsistent effects may also be attributable to the use of insensitive experimental designs (e.g. between subjects) or inappropriate selection of baseline measures and the failure to include these in the statistical analysis. Two psychological measures of mood may claim to measure the same subjective state, but it may be that only one is sufficiently sensitive to detect effects of a nutritional manipulation. It is feasible that commonly employed measures may be inadequate to assess subjective state in relation to nutrient intervention and mask real effects. A lack of effect on some measures could be due to a subtle effect of a nutrient on only one component of the subjective state which when computed into a “scale score” is no longer detected. Many studies have simply administered “off the shelf” measures and report the factor scores or summed items based on previous validation studies or factor analyses which are not based on the same populations in the same state, e.g. following a nutrition intervention.

Appropriate analyses of data from studies where subjective state is the outcome variable pose some unique problems. Even where participants can be randomised to nutrient or placebo interventions, it cannot be assumed that baseline measures will not differ and hence change from baseline or inclusion of baseline as a covariate is the recommended statistical approach. In these randomised studies, both analysis of variance (ANOVA) on change from baseline and analysis of covariance (ANCOVA) with baseline as a covariate will be unbiased [23]. In non-randomised studies where baseline differences may pre-exist and samples may be more heterogeneous, both methods may contradict each other since neither can be unbiased. True randomisation is therefore the preferred approach. Moreover, the overuse and over-interpretation of subgroup analyses poses further problems in this field [24], as does the splitting of samples using median splits [21] since these may exaggerate or even produce effects which are actually artefacts of the reduction of a continuous variable such as mood to a categorical group.

## Expectancy and placebo effects

Placebo effects are a concern for all scientific research and well-designed clinical studies go to great lengths to avoid potential bias introduced by these effects by “blinding”. However, via expectations, placebos influence a variety of outcome measures including physiological responses. Despite continuing efforts to fully understand the psychological mechanisms of placebo effects, it is well recognised that expectation is a major contributor to study outcomes in response to treatment in intervention studies [25]. Expectancy effects can be described as changes in behaviour that occur in an experiment due to the anticipation of results that unconsciously affect the outcome. The observation that these effects may be one of the main mechanisms that underlie the placebo effect has been supported by neuroimaging studies which have shown that the placebo response is associated with widespread frontal and prefrontal activation which is consistent with the processing of expectation and executive function [26]. Most of our knowledge about placebo mechanisms comes from investigations of placebo effects in the domain of pain [27] where patients have repeatedly experienced significant pain relief from placebos when the placebo is described as an analgesic medication compared to when it is not [28]. This is an example of a verbal cue that can create a strong expectancy effect which results in behavioural, clinical or physiological changes [29]. Another example of this is where caffeine-associated stimuli such as the smell and taste of coffee have been shown to elicit both self-reported and physiological arousal [30] and where the expectation of having consumed caffeine can result in improvements in cognitive performance and mood [18]. In this particular study, participants were randomly allocated to both a drink (caffeinated coffee vs. decaffeinated coffee) and an expectancy (told caffeine vs. told decaffeinated) condition. Both caffeine and expectation of having consumed caffeine improved attention and psychomotor speed.

There are many other factors which influence expectation including previous experience of the intervention, knowledge of others’ accounts, influences such as the researcher and non-verbal and sensory cues such as the label of the product and the taste. For instance, Rogers et al. [31] showed that when energy drinks were labelled “energy drink” rather than “healthy and nutritious”, both the expected energy from the drink and the expectation of better performance on cognitive tasks were significantly greater. In this study, taste also resulted in expectation of better performance on the task; however, these expectancies did not carry through to influence actual performance, which is contradictory to Green et al.’s study [32]. They did find that expectancy effects were able to influence performance on a cognitive task. Green et al. [32] gave study participants either glucose or aspartame on two occasions in a counterbalanced manner. On one occasion, for each treatment, participants were misinformed about the treatment they were going to receive (glucose or aspartame). Improvements in the Bakan vigilance task were observed after glucose consumption, but only when participants were told they were going to receive glucose. No task improvements were observed when participants thought that they were consuming aspartame. Similar effects were observed by Stollery and Christian [33] who found a subtle improvement in a task of delayed free recall when participants believed they had consumed glucose. Whilst the authors concluded that the expectancy effects were small and unlikely to be confused with glucose enhancements, they did suggest that routine collection of product beliefs should be carried out in future studies. This is particularly important when testing commercial products, on potential consumers. Whilst typically substantial effort is invested to control for placebo effects (by having a placebo), expectancy effects are also important for subjective and objective state post-ingestion. Moreover, it is these effects reinforced by repeated exposure which will guide consumers into making a decision to purchase a product. Hence, expectancy effects also need to be measured in studies of nutrient interventions. These observations also emphasise the importance of an appropriate, well-matched placebo, particularly in experiments where the product could easily be recognised by the consumer, and post-study debriefing may be useful to assess the effectiveness of blinding of the treatment conditions (although this is rarely reported in published studies). It should also be noted that use of objective measures will not cancel or even ameliorate placebo effects. Thus, the expectancy of the study participants is an important factor in the design of nutritional intervention studies.

## Relationships between subjective and objective mental performance measures

Whilst mood states are subjective in the sense that they occur in a person’s experience, manifestations of mood can also be observed objectively in physical actions, cognition and behaviour. Mood-influenced cognition is most often studied in patients with depression or those who have recovered from depression. In such studies, depression has been shown to correlate with worse performance in attention and memory tasks. The definition of clinical depression is defined by the Diagnostic and Statistical Manual of Mental Disorders, Fifth Edition (DSM V) as a “depressed mood or a loss of interest or pleasure in daily activities for more than 2 weeks, where mood represents a change from the person’s baseline”. This rather qualitative definition is made quantitative in clinical intervention studies by establishment of threshold scores in depression checklists or questionnaires (such as the Beck Depression Inventory) which measure particular levels of impaired function in social, occupational and educational settings. Due to the concrete criteria of clinical depression, comparison of depressed vs healthy participant behaviour is a useful approach for understanding mood–cognition interactions. For instance, depressed study participants demonstrate attentional negative bias towards stimuli such as words or facial expressions as compared to non-depressed participants [34] and show a slowing down of response times compared to non-depressed participants. Interestingly, researchers have shown that participants with familial history of depression, but with no prior diagnosis of depression, also exhibit attentional bias towards negative stimuli that is greater than that of participants with no familial history of depression. These “emotional bias” cognition tests have been proposed as an early detection method to identify participants with a predisposition to depression. Similarly, functional magnetic resonance imaging (fMRI) measurements of amygdala reactivity to negative stimuli appear to be greater in participants with family history of depression or carriers of putative “depression genes” such as the 5-HTTLPR short alleles or met MAO alleles [35].

With respect to healthy participants, the negative effect of poor mood on cognition has been demonstrated with similar paradigms. For example, after a stress induction to provoke negative mood, participants exhibit stronger reactions to negative stimuli reflected by altered reaction times or decision-making [36]. Poor mood has also been shown to affect attentional biases towards punishment-associated stimuli, as manifested by faster reaction time towards such stimuli. Impaired cognitive processing due to mood has also been observed via poorer performance on effortful versus automatic visual tasks in depressed subjects [37]. The above examples describe several areas where mood has an immediate effect on cognition; however, we also need to consider the direct effects of nutrients on mood and cognition and how they may not be measurable at the same time. Consideration needs to be given to the fact that there may be a temporal lag between ingestion of a nutrient and its effect on mood and cognition. For example, improvements in mood may have a motivating effect on subsequent cognitive performance. It is highly likely that effects of nutrients or dietary supplements on mood and cognition occur within different time frames and even at different doses of the nutrient and as such effects may be missed if subjective and objective measures are not considered concomitantly and independently. For example in an acute study by Scholey et al. [19], cognitive performance and self-reported mental fatigue were measured after two doses of cocoa flavanols. Both doses improved performance on the Serial Threes task, whilst only the low dose improved mood. In addition, the high acute dose improved response times in a Rapid Visual Information Processing Task (RVIP) but showed no effects on mood. It is plausible that the effects of a nutrient on subjective state in the longer term may produce acute changes in cognition or that such changes are not related temporally. Hence, it is advisable to consider acute on chronic studies and more frequent assessments to ensure that these effects and the relationships between acute and chronic effects in both subjective and objective outcomes are captured.

## Linking subjective states to mechanisms of action

Demonstration of biological plausibility is crucial when linking the effects of specific nutrients to subjective mood states; however, there is a paucity of studies which have attempted to link these two types of outcome within the same study. We will use the example of *n* − 3 PUFA to describe potential mechanisms. Animals fed a diet containing low levels of *n* − 3 PUFA display impaired emotional and cognitive behaviour [38–47]. Metabolism of monoamines [48], neuronal plasticity [49], neuroinflammation [50, 51] and stress response [52] are impaired in the brains of these animals. In humans, an impaired stress response has been repeatedly associated with low *n* − 3 PUFA [44, 53–57]. Promising data obtained in humans and animal models show that docosahexaenoic acid (DHA) dietary supplementation lowers self-reported cognitive (e.g. feeling unease and fear) and physiological (e.g. sweating) anxiety symptoms [58], cortisol response to mental stress [55, 59] and to inflammatory stimuli [60], and peripheral and brain inflammation, especially in healthy older adults [50, 61–65]. The activity of proinflammatory cytokines in the brain are involved in the development of somatic symptoms (fatigue, attention, etc.) known to alter mental performance and mood as described above [66–68]. To conclude, preclinical studies have highlighted plausible biological mechanisms relating to neurobiological systems involved in mood and cognitive disorders, possibly underlying the effects of various nutrients on subjective mood state.

## Recommendations and conclusions

In this paper, we argue that the subjective effects of foods on self-reported mood and cognition can enrich our understanding and interpretation of gold-standard “objective outcome measures” (cognitive test outcomes, e.g. reaction times, the number of words recalled and physiological markers, e.g. salivary cortisol concentrations) and should be considered both concomitantly and independently. Thus, we make the case that measures of subjective states are a valid and appropriate methodology for assessing the effects of nutritional intake on mood and mental performance. The proposed approach extends the traditional cognitive approach of using standard “objective” performance measurements to also include consumers’ subjective experiences in relation to food. Our specific recommendations are as follows:

- Consideration of both traditional mood questionnaires or the use of ecological momentary assessment (EMA) or experience sampling methods (ESM), which may more realistically capture transient mood states. Identifying which are best and most reliable under which condition.
- Use measures sufficiently sensitive to detect effects of a nutritional manipulation and to evaluate the magnitude and meaningfulness of changes demonstrated, e.g. by comparison with effects induced by drugs or alcohol or variations in mood which occur over the course of a day.
- Consider the possibility that subjective and objective measures will occur within different time frames and in response to different doses. Acute on chronic studies may help capture transient effects on mood or cognitive function which may result in longer-term measureable benefits.
- Recognition of the importance of expectancy and placebo effects when measuring subjective mood states and behaviour generally and selection of well-matched placebos.
- Demonstrating biological plausibility is crucial when linking the effects of specific nutrients to subjective mood states and behaviour.

## Abbreviations

ANCOVA: Analysis of covariance
ANOVA: Analysis of variance
DHA: Docosahexaenoic acid
EMA: Ecological momentary assessment
ESM: Experience sampling methods
fMRI: Functional magnetic resonance imaging
POMS: Profile of mood states
PUFA: Polyunsaturated fatty acid
RVIP: Rapid visual information processing
TSST: Trier social stress test
